# Trends in Statin Use 2009–2015 in a Large Integrated Health System: Pre- and Post-2013 ACC/AHA Guideline on Treatment of Blood Cholesterol

**DOI:** 10.1007/s10557-018-6810-1

**Published:** 2018-07-30

**Authors:** Teresa N. Harrison, Ronald D. Scott, T. Craig Cheetham, Shen-Chih Chang, Jin-Wen Y. Hsu, Rong Wei, Deborah S. Ling Grant, Susan H. Boklage, Victoria Romo-LeTourneau, Kristi Reynolds

**Affiliations:** 1 Kaiser Permanente Southern California, Department of Research & Evaluation, 100 S. Los Robles, 2nd Floor, Pasadena, CA 91101 USA; 2 Southern California Permanente Medical Group, West Los Angeles Medical Center, Los Angeles, CA USA; 3 Western University – College of Pharmacy, Pomona, CA USA; 4 California Maternal Quality Care Collaborative, Division of Neonatal and Developmental Medicine, Stanford University, Palo Alto, CA USA; 5 Regeneron Pharmaceuticals, Inc., 777 Old Saw Mill River Rd, Tarrytown, NY 10591 USA; 6 US Medical Affairs, Sanofi Aventis Group, Bridgewater, NJ USA

**Keywords:** Hypercholesterolemia, Statins, Prescribing trends, Practice guideline

## Abstract

**Purpose:**

Implementation of the 2013 ACC/AHA cholesterol treatment guideline is likely to vary by statin benefit group. The aim of this study was to document trends in statin use before and after introduction of the ACC/AHA guideline.

**Methods:**

We conducted a retrospective study with annual cohorts from 2009 to 2015 among members of Kaiser Permanente Southern California aged ≥ 21 years. Members were categorized into four mutually exclusive statin benefit groups: atherosclerotic cardiovascular disease (ASCVD), LDL-C ≥ 190 mg/dL in the last year, diabetes (aged 40–75 years), and 10-year ASCVD risk ≥ 7.5% (aged 40–75 years).

**Results:**

The cohorts ranged from 1,993,755 members in 2009 to 2,440,429 in 2015. Approximately 5% of patients had ASCVD, 1% had LDL-C ≥ 190 mg/dL, 6% had diabetes, and 10% had a 10-year ASCVD risk ≥ 7.5% each year. Trends in statin use were stable for adults with ASCVD (2009 78%; 2015 80%), recent LDL-C ≥ 190 mg/dL (2009 45%; 2015 44%), and diabetes (2009 74%; 2015 73%), but increased for patients with 10-year ASCVD risk ≥ 7.5% (2009 36%; 2015 47%). High-intensity statin use also increased 142% and 54% among patients with LDL-C ≥ 190 mg/dL and those with ASCVD ≤ 75 years of age, respectively. Moderate-to-high intensity statin utilization increased over 50% among those with a 10-year ASCVD risk ≥ 7.5%.

**Conclusions:**

Statin use increased substantially among patients with 10-year ASCVD risk ≥ 7.5% and use of appropriate statin dosage increased in each of the four statin benefit groups between 2009 and 2015; however, there is room for improvement.

## Introduction

In 2013, the American College of Cardiology (ACC) and the American Heart Association (AHA) released a new guideline on the treatment of high blood cholesterol to reduce the development of atherosclerotic cardiovascular disease (ASCVD) [1]. The guideline supplants the Adult Treatment Panel III guideline which recommended treating to specific low-density lipoprotein cholesterol (LDL-C) targets [1, 2] and expanded treatment to all adult patients with known ASCVD, regardless of LDL-C levels. For primary prevention, the guideline expanded treatment to individuals in the following statin benefit groups: (1) LDL-C ≥ 190 mg/dL; (2) diabetes aged 40–75 years and LDL-C 70–189 mg/dL; or (3) an estimated 10-year risk of ASCVD ≥ 7.5%, aged 40–75 years and LDL-C 70–189 mg/dL.

A systematic review of randomized clinical trials was used to support the new ACC/AHA guideline [1] and found that treating patients in each benefit group required appropriate statin dosage intensity to achieve a specific therapeutic response. Subsequently, the National Committee for Quality Assurance proposed Healthcare Effectiveness Data and Information Set metrics based on the ACC/AHA guideline. The proposed quality metric assesses the percentage of patients with clinical ASCVD or diabetes who were dispensed a statin during the measurement year and who remained on a statin medication of any intensity for at least 80% of the treatment period [3].

Studies which have examined the effect of the 2013 ACC/AHA guideline on statin treatment patterns have found increased use of high-intensity statins among patients with ASCVD in the post-guideline period [4–7]; however, implementation of the ACC/AHA guideline is likely to differ by healthcare setting and by statin benefit group. Therefore, assessing statin treatment patterns can provide clinicians and administrators with insights and opportunities to increase statin use and ultimately decrease ASCVD events. The overall objective of the current study was to document trends in statin use and appropriate statin intensity before and after publication of the 2013 ACC/AHA guideline among adults in a large and diverse integrated healthcare delivery system.

## Methods

### Setting and Study Population

This study was conducted among members of Kaiser Permanente Southern California (KPSC), an integrated healthcare delivery system that currently provides care for more than 4.5 million members. The KPSC population is highly representative of the Southern California region except for slightly lower representation at the extremes of income and education [8]. Data on medical care are captured through structured administrative and clinical databases and an electronic health record (EHR). Most KPSC members (> 95% in recent years) have a drug benefit and have their prescriptions filled at KPSC pharmacies. KPSC members aged ≥ 21 years on September 30 (index date) of each study year (2009–2015), with continuous membership and a continuous pharmacy benefit (administrative gap ≤ 45 days allowed) 12 months prior to the index date, were eligible for inclusion. After exclusions, the number of members in each cohort year gradually increased from 1,993,755 in 2009 to 2,440,429 in 2015.

### ACC/AHA Statin Benefit Groups

**Fig. 1:**
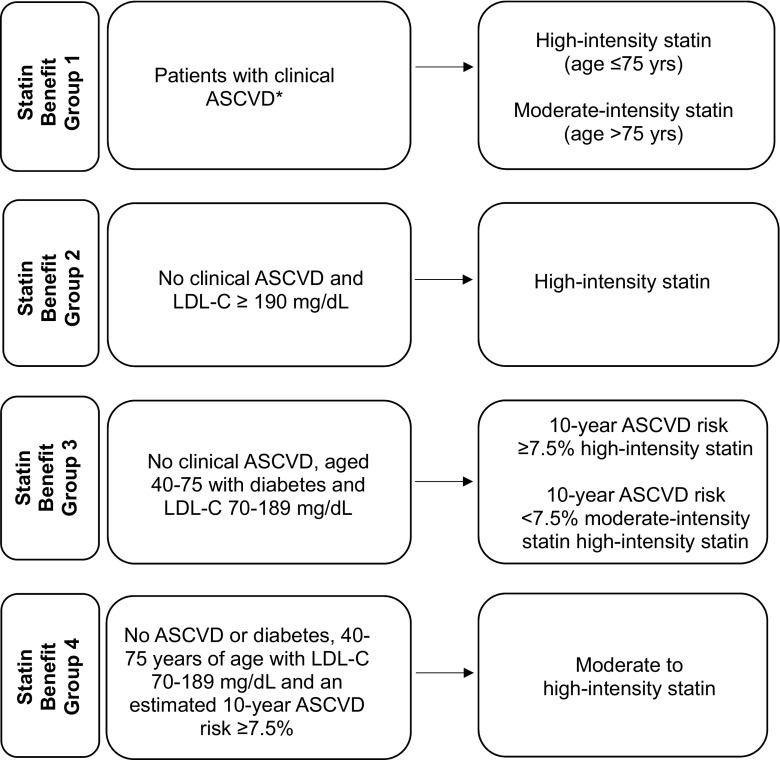
Statin benefit group and statin intensity recommendation. ASCVD is defined as acute coronary syndromes, a history of myocardial infarction, stable or unstable angina, coronary or other revascularization, and stroke or peripheral arterial disease of atherosclerotic origin (asterisk)

As shown in Fig. 1, patients were categorized in each cohort year into the four mutually exclusive statin benefit groups in the recommended hierarchical order ((1) clinical ASCVD, (2) recent LDL-C ≥ 190 mg/dL, (3) diabetes age 40–75 years, and (4) an estimated 10-year ASCVD risk score ≥ 7.5%). Clinical ASCVD was defined as acute coronary syndrome, history of myocardial infarction, stable or unstable angina, coronary or other revascularization procedures, stroke, or peripheral arterial disease of atherosclerotic origin based on *International Classification of Diseases, Ninth Revision, Clinical Modification (ICD-9)* diagnosis, and procedure codes. The second statin benefit group included patients with LDL-C ≥ 190 mg/dL *within 12 months* prior to the index date and no past medical history of ASCVD. The third statin benefit group included patients 40–75 years of age with a history of type 1 or type 2 diabetes defined as any of the following in the 12 months prior to the index date: at least one primary inpatient discharge diagnosis of ICD-9 250.x, two or more outpatient diagnoses of ICD-9 250.x occurring on separate dates within 12 months prior to the index date, or one or more dispensed prescriptions for insulin or an oral hypoglycemic agent (patients only using metformin and without a diagnosis of diabetes were excluded from the definition of diabetes). Women diagnosed with gestational diabetes within 12 months of the index date were also excluded from the definition of diabetes. The fourth statin benefit group included patients without ASCVD or diabetes who were 40–75 years of age with LDL-C 70–189 mg/dL and an estimated 10-year ASCVD risk ≥ 7.5% calculated using the Pooled Cohort ASCVD Risk Equations [9]. Although the 10-year ASCVD risk calculator is valid for patients 40–79 years of age, the ACC/AHA treatment guideline does not have strong recommendations for statin use among those aged > 75 years without ASCVD, LDL-C ≥ 190 mg/dL or diabetes; thus, they were excluded from the fourth statin benefit group [1]. Patients were excluded from the study if they had any of the following contraindications to statins within 12 months prior to the index date: pregnancy, in vitro fertilization, use of clomiphene, end-stage renal disease, or cirrhosis. Patients in hospice or a skilled nursing facility or those who had a history of rhabdomyolysis were also excluded from the study.

### Baseline Patient Characteristics

Baseline characteristics of the study population were defined using data in the 12 months prior to and including the index date. These included sociodemographics (age, sex, and race/ethnicity), Charlson Comorbidity Index (CCI) (which includes myocardial infarction, peripheral vascular disease, cerebrovascular disease, and diabetes) [10], LDL-C, and health plan type (Commercial, Medi-Cal, Medicare, and private pay). Use of statin medication (including combination therapy) was identified by the dispense date of the prescription and was assessed in the 12 months prior to the index date. Intensity of statin therapy was assigned according to the ACC/AHA guideline [1].

To estimate patients’ 10-year risk of developing a first ASCVD event, the following variables were included in the Pooled Cohort ASCVD Risk Equations: age, total cholesterol, high-density lipoprotein cholesterol, treatment for hypertension, systolic blood pressure, current smoking status, diabetes, sex, and race (African-American or white) [9]. Patients missing data to calculate ASCVD risk were excluded from this analysis (20–25% each year).

### Statistical Analysis

Patients were categorized into the four statin benefit groups for each year of the study (2009–2015), and the percentage with a dispensed statin prescription in the 12-months prior to the index date was calculated. Baseline patient characteristics were examined by statin use (users vs. non-users) across study years for each of the four statin benefit groups. Next, the percentage of patients in each statin benefit group with the appropriate statin intensity was calculated. Statin intensity was assessed separately for patients ≤ 75 and > 75 years of age in the ASCVD statin benefit group, those with diabetes and ASCVD risk < 7.5% and ≥ 7.5%, and for patients with ASCVD risk 7.5–14.9% and ≥ 15% per the ACC/AHA treatment recommendation [1]. Adherence to statin medication was calculated for the overall study population in each year using the proportion of days covered (PDC) calculated during the 12 months prior to the index date divided by the number of days covered (based on prescription dispense dates and days of supply). Adherence was defined as a PDC ≥ 80% [11].

The study protocol was reviewed and approved by the KPSC institutional review board, and a waiver for written informed consent was obtained due to the retrospective nature of the study. Compliance with Health Insurance Portability and Accountability Act regulations was ensured.

## Results

Among the nearly 2 million or more patients included in each study year, approximately 5% had ASCVD, 1% had LDL-C ≥ 190 mg/dL, 6% had diabetes, and 10% had a 10-year ASCVD risk ≥ 7.5% (Table 1). The proportion of patients in each statin benefit group and the distribution of patient characteristics among statin users and non-users was stable across years; therefore, only data for 2009 and 2015 are reported. In the ASCVD statin-benefit group, mean age was similar among statin users vs. non-users across all years; however, a higher proportion of statin users compared to non-users were male (64 vs. 52–53%), Medicare beneficiaries (61–67 vs. 52–57%), and had substantially lower mean LDL-C (80–86 vs. 109–111 mg/dL). Statin users in the ASCVD group also had higher health care utilization and higher CCI scores compared to statin non-users. Fewer non-Hispanic black patients with ASCVD were statin users vs. non-users (approximately 10 vs. 13%), while statin use increased among ASCVD patients with CCI ≥ 3 during the study period. Among patients in the LDL-C ≥ 190 mg/dL statin benefit group, statin users were slightly older, more likely to be non-Hispanic black or Hispanic, to have ≥ 6 ambulatory visits, and to have more comorbidities than those patients not using statins. Mean LDL-C was slightly higher for statin users vs. non-users (212 vs. 209 mg/dL) across the years. Statin users with diabetes and without ASCVD or LDL-C ≥ 190 mg/dL were older, had more ambulatory care utilization, and a CCI score ≥ 3 than patients not using statins (30 vs. 23%). A larger proportion of statin users with diabetes had Medicare coverage (24–33%) than patients not on statins (16–22%). Patients with a 10-year ASCVD risk score ≥ 7.5% and using statins were likely to be older, female, and had higher ambulatory care utilization and ≥ 3 comorbidities compared to non-statin users (8–11 vs. 4–6%).

**Table 1:** Baseline characteristics of the KPSC population by statin benefit group, years 2009 and 2015

	ASCVD and age ≥ 21 years				LDL-C ≥ 190 mg/dL and age ≥ 21 years				Diabetes, LDL-C 70–189 mg/dL and age 40–75 years				ASCVD risk ≥ 7.5%, LDL-C 70–189 mg/dL and age 40–75 years			
Year	2009		2015		2009		2015		2009		2015		2009		2015	
Total eligible population	1,993,755		2,440,429		1,993,755		2,440,429		1,993,755		2,440,429		1,993,755		2,440,429	
Eligible for statin use within benefit group	105,186 (5.3)		116,439 (4.8)		16,922 (0.8)		14,659 (0.6)		113,670 (5.7)		145,759 (6.0)		212,599 (10.7)		244,578 (10.0)	
	No	Yes	No	Yes	No	Yes	No	Yes	No	Yes	No	Yes	No	Yes	No	Yes
Statin use, %	22.2	77.8	20.2	79.8	55.5	44.5	56.3	43.7	22.6	77.4	20.8	79.2	63.8	36.2	53.4	46.6
Mean age (SD)	68.8 (13.9)	70.3 (10.9)	70.1 (14.0)	71.9 (10.8)	52.9 (13.1)	55.3 (11.5)	53.6 (13.7)	56.4 (11.9)	56.4 (9.1)	59.2 (8.8)	57.7 (9.4)	60.9 (8.7)	63.7 (7.3)	65.9 (6.4)	65.5 (6.4)	67.6 (5.4)
Sex, %																
Female	47.0	36.4	48.1	35.8	57.7	59.0	59.3	59.2	48.8	49.5	53.0	49.1	31.5	36.7	33.7	36.8
Male	53.0	63.6	51.9	64.2	42.3	41.0	40.7	40.8	51.2	50.5	47.0	50.9	68.5	63.3	66.3	63.2
Race/ethnicity, %																
White, non-Hispanic	58.6	60.6	56.2	56.6	42.3	38.1	45.5	38.2	29.0	32.2	26.8	29.3	49.4	53.7	49.8	51.4
Black, non-Hispanic	12.9	10.6	13.0	10.0	12.4	16.1	10.7	12.8	13.9	13.1	14.0	11.5	15.4	13.5	14.0	11.2
Asian or Pacific Islander	5.9	7.9	6.7	9.6	8.2	8.5	9.2	10.6	9.6	13.0	9.7	14.4	8.4	9.9	8.5	11.4
Hispanic	19.5	19.0	22.0	22.2	29.5	31.4	29.2	34.2	42.6	37.5	46.3	41.7	21.6	19.0	23.9	22.8
Multiple/other/unknown	3.1	1.9	2.1	1.5	7.7	5.9	5.3	4.2	5.0	4.2	3.3	3.1	5.2	3.9	3.8	3.2
Ambulatory visits, %																
0	9.1	1.5	9.2	1.6	2.2	1.2	2.6	1.6	3.7	1.4	3.2	1.6	6.9	2.5	7.5	2.6
1–5	29.2	24.2	29.8	25.3	60.0	53.9	60.5	54.9	36.9	33.1	37.1	34.6	50.2	44.1	51.0	46.0
≥ 6	61.7	74.3	61.0	73.2	37.9	44.8	36.9	43.5	59.4	65.5	59.7	63.8	42.9	53.4	41.5	51.4
Charlson Comorbidity Score, %																
0	36.9	17.6	3.5	13.0	77.6	67.8	73.0	62.5	5.9	3.0	5.6	3.5	74.9	62.4	67.8	49.0
1	19.7	20.6	19.2	18.5	13.2	17.7	15.6	21.2	47.3	44.3	45.3	40.2	13.4	17.7	17.1	25.4
2	14.9	17.0	15.3	17.4	5.6	8.6	6.2	9.0	23.8	23.8	26.0	26.0	7.6	12.2	9.2	14.4
≥ 3	28.5	44.7	35.0	51.0	3.6	5.9	5.2	7.4	23.0	28.9	23.1	30.3	4.1	7.7	5.9	11.2
Health plan type, %																
Commercial	45.0	36.9	38.7	30.3	79.4	78.7	72.9	70.3	80.3	72.5	72.3	61.7	60.1	48.4	49.6	37.2
Medicaid	1.2	0.5	2.0	1.1	0.7	0.7	2.7	3.7	1.4	1.0	3.2	2.8	0.4	0.3	1.1	0.8
Medicare	51.7	60.9	57.3	67.0	14.5	15.9	18.2	21.1	15.9	24.0	21.6	32.7	36.1	48.8	46.4	60.1
Private pay	2.1	1.7	1.9	1.6	5.5	4.7	6.2	4.9	2.4	2.5	2.9	2.7	3.3	2.5	2.8	1.8
Mean LDL-C (SD)	111.0 (35.7)	85.5 (28.6)	109.1 (37.0)	80.3 (28.1)	208.8 (22.0)	212.0 (24.4)	208.7 (24.5)	212.9 (26.4)	100.8 (30.7)	86.8 (28.9)	102.1 (32.5)	82.0 (28.8)	126.3 (30.1)	108.6 (31.4)	120.6 (31.4)	97.4 (31.9)

Data shown are *N* (percentage) or mean (standard deviation)

**Fig. 2:**
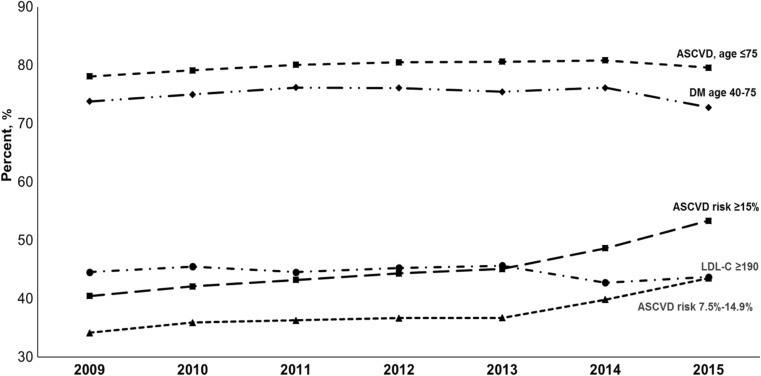
Percentage of patients in Kaiser Permanente Southern California using statins by statin benefit group, 2009–2015. (1) Clinical ASCVD; (2) LDL-C ≥ 190 mg/dL; (3) diabetes aged 40–75 years and LDL-C 70–189 mg/dL; and (4) no ASCVD or diabetes, 40–75 years of age with LDL-C 70–189 mg/dL and an estimated 10-year ASCVD risk ≥ 7.5%. DM diabetes mellitis, ASCVD atherosclerotic cardiovascular disease, LDL-C low-density lipoprotein cholesterol

The prevalence of statin treatment by statin benefit group is shown in Fig. 2. Among patients eligible for statin therapy, use of statins was stable over time for adults with ASCVD age ≤ 75 years (2009 78%; 2015 80%), LDL-C ≥ 190 mg/dL (2009 45%; 2015 44%), and diabetes (2009 74%; 2015 73%). The prevalence of statin use increased gradually for the 10-year ASCVD risk 7.5%–14.9% group between 2009 (34%) and 2013 (37%) then rose substantially in 2015 (44%). Similarly, the prevalence of statin use increased in the 10-year ASCVD risk ≥ 15% group from 41% in 2009 to 45% in 2013 and then to 53% in 2015.

**Fig. 3:**
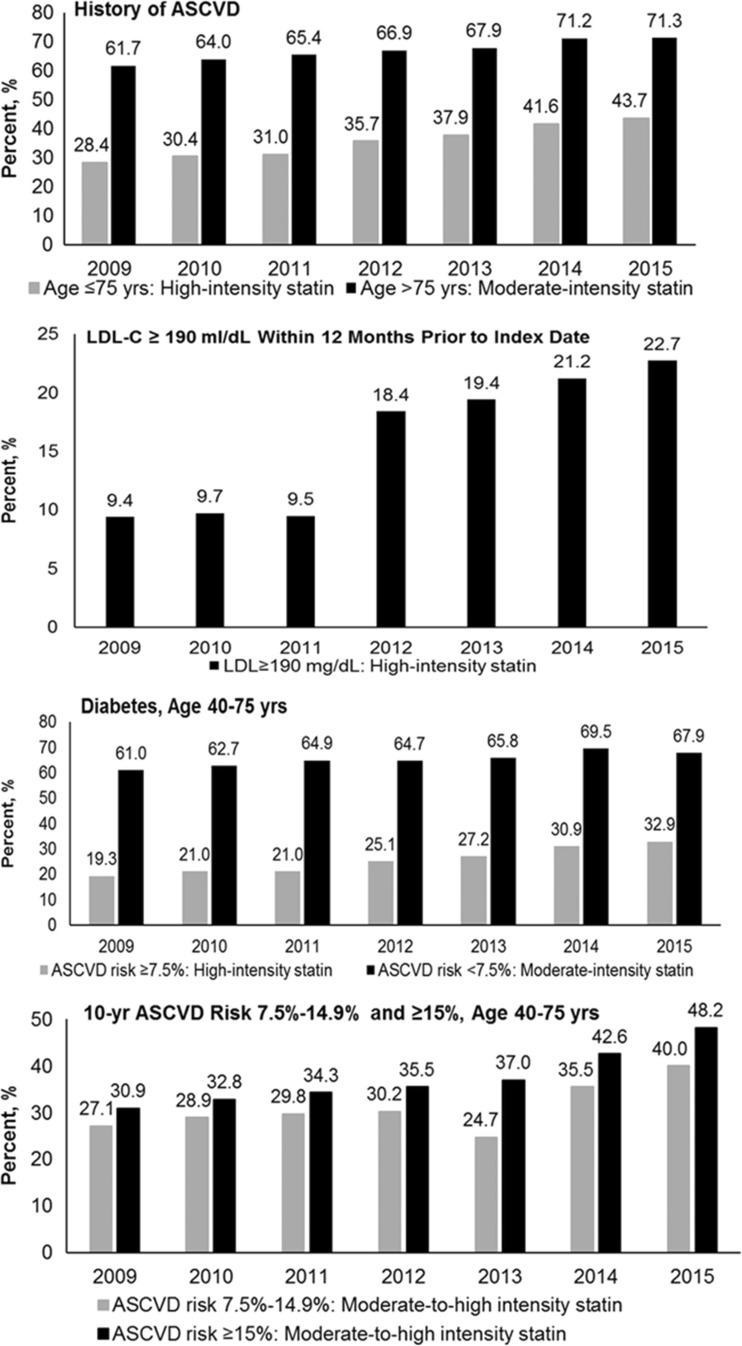
Percentage of patients in Kaiser Permanente Southern California with appropriate statin intensity use by statin benefit group, 2009–2015

The prevalence of appropriate intensity statin use increased over time in all four benefit groups (Fig. 3). High-intensity statin use increased 142% from 2009 to 2015 among patients with LDL-C ≥ 190 mg/dL, followed by those with diabetes and ASCVD risk ≥ 7.5% (70%) and those with ASCVD who were less than 75 years of age (54%). Appropriate use of moderate-intensity statins increased by 11% among those with diabetes and ASCVD risk < 7.5%, and by 16% among patients > 75 years with ASCVD. Moderate-to-high intensity statin utilization increased by 48 and 56%, respectively, among those with a 10-year ASCVD risk 7.5–14.9 and ≥ 15%.

The mean PDC was uniform across all study years with each cohort having 84% of their treatment time covered by statin use. Nearly 70% of patients were considered adherent to statin therapy (PDC ≥ 80%) during the year prior to the index date.

## Discussion

The current study found that statin use among patients in the ASCVD and diabetes statin benefit groups remained stable (> 75%); however, the prevalence of treatment among patients with LDL-C ≥ 190 mg/dL in the year prior to the index date was lower than 50% before and after publication of the 2013 ACC/AHA cholesterol treatment guideline. Treatment among patients with a 10-year ASCVD risk score ≥7.5% rose steadily beginning in 2013. There was some variation in patient characteristics between statin users and non-users. In each of the benefit groups, statin users had more ambulatory visits and co-morbidities. Fewer non-Hispanic Black patients with ASCVD were statin users, while the treatment rate increased among Hispanics during the study period.

The proportion of patients with appropriate statin use increased from 2009 to 2015 in each of the four statin benefit groups with a higher proportion of patients using moderate-intensity statins according to the recommended treatment guideline. Although there were substantial increases in appropriate high-intensity statin use from 2009 to 2015, 56% of patients with ASCVD and 67% of patients with diabetes and ASCVD risk ≥ 7.5% were not being treated in 2015 at goal thus indicating opportunities to improve provider knowledge regarding the benefits of high-intensity statins [12]. Despite having a high lifetime risk of experiencing an ASCVD event, a low proportion of patients in the LDL-C ≥ 190 mg/dL benefit group were dispensed a high-intensity statin. And, although moderate-to-high intensity statin use increased significantly for adults with 10-year ASCVD risk 7.5–14.9%, approximately 135,000 eligible KPSC patients were not receiving statins post-ACC/AHA guideline.

Using an administrative claims database, Steen et al. performed an analysis of prescription patterns of statins and non-statin lipid lowering medications among more than 1 million individuals with ASCVD or diabetes in 2014 [13]. Among patients with ASCVD, 50% filled a prescription for a lipid lowering medication, while only 40% of those with diabetes filled such a prescription. Only 27% of patients with ASCVD and 7% of those with diabetes were taking a high-intensity statin. Another analysis using a claims database among patients with ASCVD showed that use of high-potency statins increased from 13% in 2004 to 26% in 2012 [14]. Rosenson and colleagues assessed trends in high-intensity statin use following hospitalization for myocardial infarction using administrative claims data between 2011 and 2014 [15]. The overall use of high-intensity statins following hospital discharge increased more than two-fold over the study period to 72 and 58%, respectively, among those < 65 and 66–75 years of age. Findings from the 2011–2012 National Health and Nutrition Examination Survey (NHANES) showed that statin use among the four statin benefit groups was 64% for the ASCVD group, 61% for LDL-C ≥ 190 mg/dL, 43% for diabetes, and 27% for 10-year ASCVD risk ≥ 7.5% [16]. The present study findings demonstrate a similar or higher prevalence of statin use and appropriate statin intensity compared to studies conducted previously.

Studies designed to assess the effect of the 2013 ACC/AHA guideline on statin treatment patterns and adherence also found suboptimal utilization of statins, although high-intensity statin use generally increased following publication of the guideline [4–7]. Bellows et al. matched a post-guideline cohort to a historical cohort on pre-index antihyperlipidemic medication classification (non-statin therapy or low, moderate, or high intensity statin) in a post hoc analysis among ASCVD patients in a regional managed care organization [4]. More patients in the post-guideline cohort (24%) were receiving high-intensity statins compared to the historical group (16%), while use of non-statin lipid lowering medications decreased. In addition, adherence assessed via PDC was higher among patients in the post-guideline group (67%) vs. the historical cohort (57%). Among mostly commercially insured patients covered by a national health plan, high-intensity statin use increased across the highest risk groups (ASCVD, LDL-C ≥ 190 mg/dL and diabetes with a 10-year ASCVD risk ≥ 7.5%) after guideline implementation, which is similar to the present study findings [6]. Two studies found no change or a decline in overall statin use rates in the year following the publication of the cholesterol guideline; however, there were modest increases in the proportion of ASCVD and diabetes patients on high-intensity statins following the 2013 guideline introduction [5, 7]. Pokharel and colleagues conducted a study among patients from 161 cardiology practices participating in the Practice Innovation and Clinical Excellence (PINNACLE) Registry and found a 4% increase in moderate-to-high-intensity statin use among patients with ASCVD following adoption of the ACC/AHA guideline [17]. There was no change in the other three statin benefit groups. These findings demonstrate limited implementation of the 2013 guideline despite estimations of increased eligibility of patients for statin therapy and subsequent utilization [18, 19]. The differences in treatment rates and appropriate use of high-intensity statins vary by study and may be a result of quality-improvement strategies which may be more routinely implemented in an integrated healthcare delivery system and/or clinicians’ knowledge of the recommended treatment for different statin benefit groups. A recent survey among a sample of 513 providers found several knowledge gaps in their understanding of the 2013 guideline [20]. For example, only 51% of providers in training and 47% in practice were able to identify the four statin benefit groups and 36% of providers in training and 48% of those in practice could identify a patient with familial hypercholesterolemia. Furthermore, lack of familiarity with the guideline was cited by 34% of surveyed providers as a major barrier to use of the guideline.

This study has several limitations. First, data were limited to a single health plan where most patients have a drug benefit and the convenience of accessing the pharmacy at their medical center. Therefore, the findings may not be generalizable to other populations, such as uninsured populations. Second, no direct contact was made with patients or healthcare providers to determine the reason for not receiving or filling a statin prescription; therefore, challenges to meeting treatment recommendations for patients with ASCVD and those at high risk of cardiovascular disease are not determined. For example, some patients may be statin intolerant and therefore may not receive a statin prescription or decline to fill a statin prescription. Further, a clinician may not be familiar with the ACC/AHA guideline and thus not prescribe statins accordingly. The primary advantage of this study is that it was conducted in a large, diverse population using EHR and administrative data which provide complete information about patients at the point of care compared to solely using administrative claims data. In addition, trends are reported over several years and across all four statin benefit groups. Lastly, initiatives implemented within KPSC (e.g., provider and staff education, monitoring and feedback, decision support, automated telephone reminders, and online engagement) in addition to the ACC/AHA guideline may have influenced statin treatment rates and adherence.

In conclusion, statin use and use of high-intensity statins in this health system trended up from 2009 to 2015. These rates of statin utilization may be higher than what is seen in other health care settings; nonetheless, there is room for improvement to further reduce cardiovascular events, as the use of high-intensity statins was lower than expected in a health care system that encourages use of appropriate statin dosage intensity.

Examining the association between appropriate statin use and cardiovascular outcomes would provide additional information to support development of strategies for management of high-risk patients.
